# Latent class analysis of behavior across dog breeds reveal underlying temperament profiles

**DOI:** 10.1038/s41598-022-20053-6

**Published:** 2022-09-17

**Authors:** Isain Zapata, Alexander W. Eyre, Carlos E. Alvarez, James A. Serpell

**Affiliations:** 1 Department of Biomedical Sciences, Rocky Vista University, 8401 S. Chambers Rd., Parker, CO 80112 USA; 2 Center for Clinical and Translational Research, The Abigail Wexner Research Institute at Nationwide Children’s Hospital, Columbus, OH USA; 3 Departments of Pediatrics and Veterinary Clinical Sciences, The Ohio State University Colleges of Medicine and Veterinary Medicine, Columbus, OH 43210 USA; 4 School of Veterinary Medicine, University of Pennsylvania, Philadelphia, PA USA

**Keywords:** Animal behaviour, Functional clustering

## Abstract

Latent class analysis (LCA) is a type of modeling analysis approach that has been used to identify unobserved groups or subgroups within multivariate categorical data. LCA has been used for a wide array of psychological evaluations in humans, including the identification of depression subtypes or PTSD comorbidity patterns. However, it has never been used for the assessment of animal behavior. Our objective here is to identify behavioral profile-types of dogs using LCA. The LCA was performed on a C-BARQ behavioral questionnaire dataset from 57,454 participants representing over 350 pure breeds and mixed breed dogs. Two, three, and four class LCA models were developed using C-BARQ trait scores and environmental covariates. In our study, LCA is shown as an effective and flexible tool to classify behavioral assessments. By evaluating the traits that carry the strongest relevance, it was possible to define the basis of these grouping differences. Groupings can be ranked and used as levels for simplified comparisons of complex constructs, such as temperament, that could be further exploited in downstream applications such as genomic association analyses. We propose this approach will facilitate dissection of physiological and environmental factors associated with psychopathology in dogs, humans, and mammals in general.

## Introduction

The Canine Behavioral Assessment and Research Questionnaire (C-BARQ) was created as a quantitative tool originally designed to measure the prevalence and severity of behavioral problems in privately-owned and working dogs^1^. This tool works by performing a wide assessment of problematic behavior from the point of view of the owner or handler and can be utilized by pet owners, working dog handlers, and clinicians to identify behavioral problems^2,3^. The C-BARQ assessment tool has been widely used to characterize dog behavior on population aggregates that are unique to specific geographic regions^4–6^ and behavioral management practices^7,8^. Characterization of the etiology of these problematic traits has been explored extensively using regression models and factor analysis^6,9,10^.

These approaches have allowed us to explore the dimensionality and population distribution of individual and grouped temperament traits in dogs, which provides an explanation of the physiological origins of these behaviors^11^. Other classification methods are available that provide a different view such as factorial and regression analysis that are often used to define personality types^12^. Latent class analysis (LCA) is a type of modeling analysis approach that is used to classify unobserved groups or subgroups within multivariate categorical data^13^. These tools analyze the structure of relationships between scored variables, which can be binary or multinomial, and characterizes them into latent classes. Such latent classes are characterized by a profile of conditional probabilities that define the chance of each observation being grouped within a class^14,15^.

LCA has been used for a wide array of psychological evaluations in humans, including identifying subtypes of depression^16^ or assessing PTSD comorbidity patterns^17^ or student engagement with school^18^. While LCA has been used in infectious disease epidemiology in dogs (e.g.,^19^), this is the first LCA usage in dog behavior. Specifically, we use LCA to identify a dog’s behavioral profile based on the rich and well validated C-BARQ assessment tool. This method may allow the dog research community to generate canine temperament profiles that can be used to subcategorize populations with the overarching goal of clarifying environmental and physiological cues associated with specific behaviors.

## Results

Latent class analysis (LCA) was used to define dog temperament profiles derived from the C-BARQ assessment tool. Notably, the C-BARQ primarily measures problematic behavioral traits. These are coded in a Likert scale from 0 to 4 where higher C-BARQ scores are indicative of the behavior being less desirable. The effect of the covariates in the C-BARQ LCA models is presented in Table 1. It is important to keep in mind that every time the modeling is run, the assigned numbering for each class is assigned arbitrarily within the selected number of classes. All covariates displayed a significant relevance in the models with strong P-values. However, by assessing of the difference between the total log-likelihood values for the models with and without covariates, we see that the contribution of covariates is small (2-class model, − 2,018,389.15 with and − 2,096,771.89 without covariates, 3.74% contribution; 3-class model, − 1,998,480.14 with and − 2,076,735.29 without covariates, 3.77% contribution; 4-class model, − 1,983,779.49 with and − 2,064,066.2 without covariates, 3.89% contribution). Model fit data (AIC, BIC, CAIC and ABIC) for the 2-, 3- and 4-class models with and without covariates are available as Supplementary Table S1. Overall, model fit showed very consistent small improvements in fit across all statistics indicating that models with a larger number of classes have a better fit. Our approach in this study stopped after 4 classes even though a larger number of classes would provide a better fit. Effect sizes for the covariates can be difficult to interpret although they suggest interesting possibilities. For example, neutered status has a larger effect size than sex. Also, the effect of having multiple dogs in the same household is larger than the health of the individual dog studied.

**Table 1:** Odds ratio effect size estimated for covariates in the models.

Class	2 class model			3 class model				4 class model				
	p-value	1	2	p-value	1	2	3	p-value	1	2	3	4
Intercept		Reference	0.654		Reference	0.373	0.698		Reference	5.401	2.645	1.326
Sex (male)	< 0.0001		0.823	< 0.0001		0.926	1.293	< 0.0001		1.203	1.405	0.997
Age at evaluation	< 0.0001		1.001	< 0.0001		1.002	1.000	< 0.0001		0.994	0.997	0.999
Weight (kg)	< 0.0001		1.014	< 0.0001		1.008	0.984	< 0.0001		1.006	0.989	1.013
Neutered status (yes)	< 0.0001		0.686	< 0.0001		0.608	0.983	< 0.0001		0.530	0.635	0.389
Age acquired (weeks)	0.0052		1.000	< 0.0001		1.001	1.001	< 0.0001		0.994	0.999	0.999
Health Problems (yes)	< 0.0001		0.846	< 0.0001		0.961	1.286	< 0.0001		0.785	1.103	0.828
First owned (yes)	< 0.0001		0.787	< 0.0001		0.690	0.956	< 0.0001		1.141	1.109	0.780
Other dogs in the household (yes)	< 0.0001		1.362	< 0.0001		1.626	1.061	< 0.0001		0.613	0.751	1.218

Estimates are presented separately. For continuous variables, estimates are for a one-unit increase.

**Figure 1:**
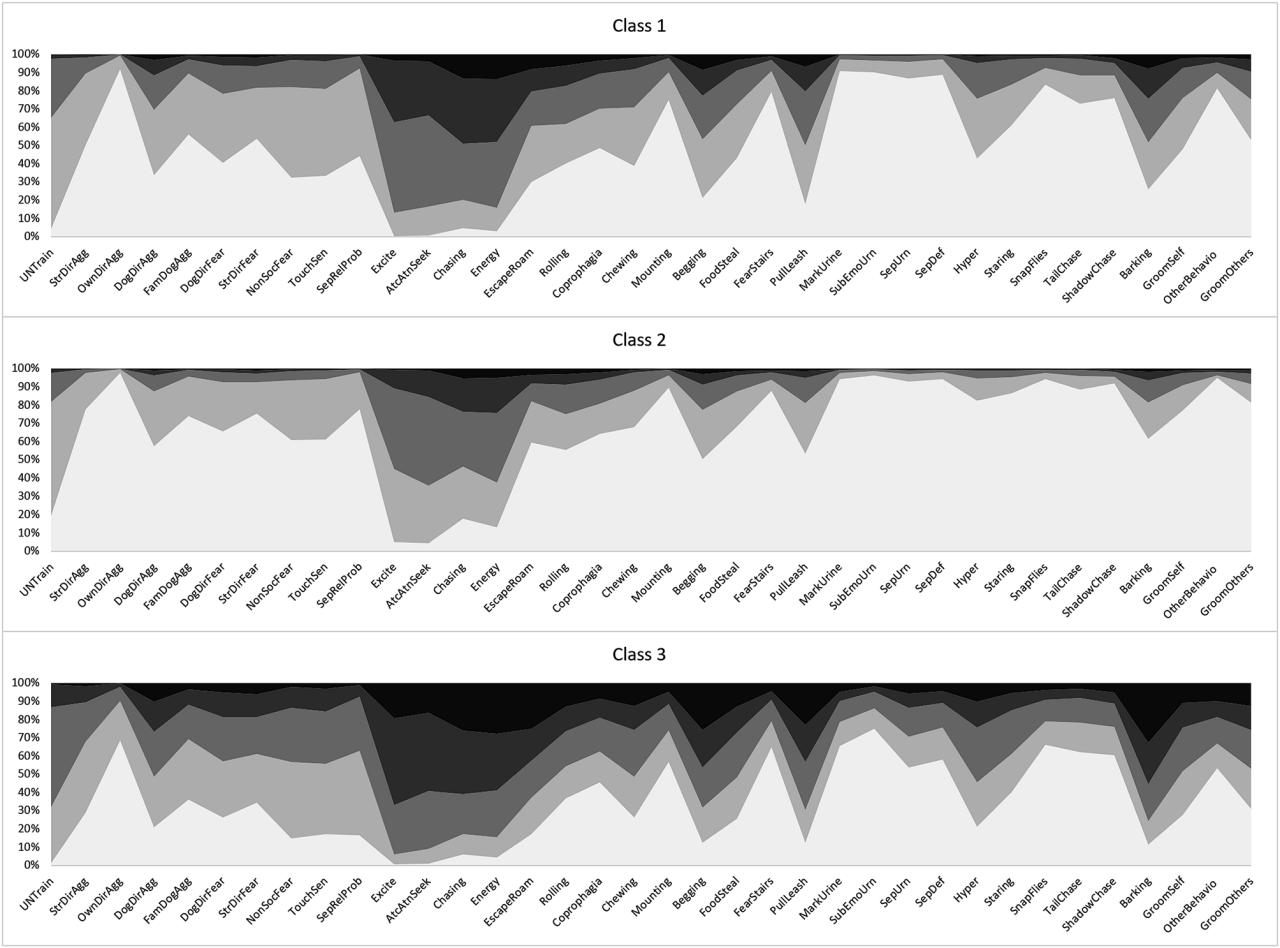
Item response probability profiles for CBARQ traits (3-class model). Class order numbering is arbitrary. CBARQ responses are coded in an ordered 5 level response scale. More desirable levels are colored in this figure with lighter shades while more undesirable levels are colored in darker shades. Using this scheme classes with more desirable CBARQ patters will display less of the darker levels displayed.

**Figure 2:**
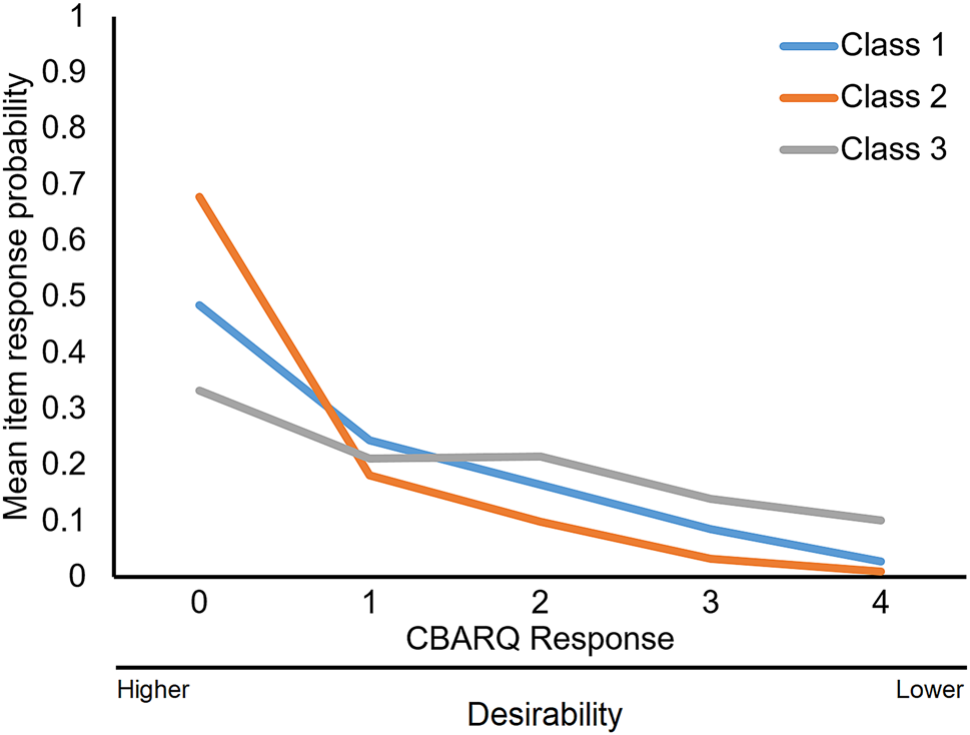
Mean response probability for the 3-class model example. This plot defines desirability for the class. Having the class with the highest proportion of the desired scores being designated as the most desirable.

LCA assigns estimated probability values to each response level for each parameter included in the model (scores within C-BARQ traits). It is thus possible to display a profile pattern for each class. In Fig. 1 we present profile pattern examples for the 3-class model which we picked for being convenient for showcasing the utility of the LCA approach. Each panel displays the probability response profile for each class. Darker shading indicates a larger proportion of less desirable scores. With these profiles, it is possible to establish the human desirability of each of the classes. In this example, Class 2 has the lowest proportion of severe scores, Class 3 the largest proportion of severe scores, and Class 1 is intermediate. This desirability pattern can be further corroborated with the mean response probabilities for each of the classes. That plot for the 3-class model is presented in Fig. 2. The line pattern which corresponds to Class 2 displays a higher proportion of mean values for the 0 level of the C-BARQ response scores (the most desirable). In contrast, Class 3 displays the lowest proportion of the most desirable “0” level scores for the C-BARQ but a much higher proportion for levels with lower desirability (3 and 4). Figures 1 and 2 support and complement each other to define desirability.

**Figure 3:**
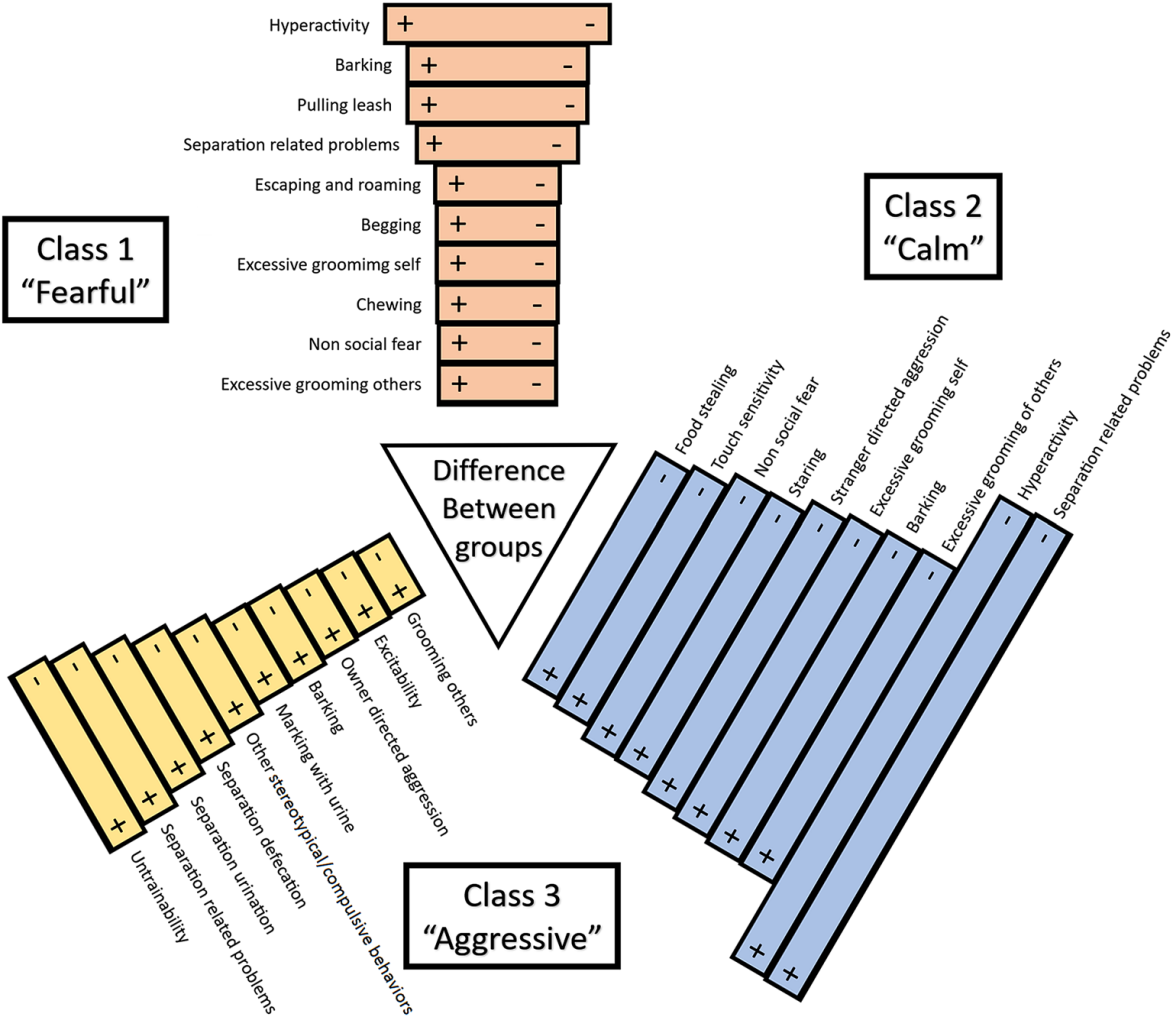
Personality profiles difference diagram for the 3-class model. These personality profiles are defined by the 10 top ranked traits based on their probability estimate pairwise differentials. The size of the bars is proportional to the square difference value for the trait. Plus (+) and minus (−) signs indicate the direction of the severity of the trait according to each pairwise comparison.

Continuing with the example of the 3-class model and using the desirability established with the profile patterns and the mean response plot (Figs. 1 and 2), we can further explore the source of these class groupings in terms of their trait differences. In Fig. 3 we present a diagram of class and trait comparisons. This diagram presents the difference between the groups as defined by the squared difference for each pairwise class comparison done for the item response probabilities. This square value quantitatively measures the probability proportion for each trait that can be used to rank them. In the diagram, the top 10 most relevant traits are presented based on these values. Additionally, a subjective descriptor label was given to each category using these top traits to associate the type of personality to a simpler construct. When comparing the most desirable group (Class 2, labeled as “Calm”) with the least desirable group (Class 3, labeled as “Aggressive”), we observe a main contrast in separation-related problems, hyperactivity, and excessive grooming of others, among other traits. This group comparison (Class 2 vs Class 3) displayed the largest difference, as suggested by its larger square difference value across all pairwise comparisons and was the only one to display any fear and aggression related traits together in their top 10 contributing traits (thus the calm and fearful labels). On a different note, when comparing the most desirable (Class 2, labeled as “Calm”) against the intermediate (Class 1, labeled as “Fearful”), the extent of the differences was smaller. Hyperactivity and barking were the top traits contributors.

**Figure 4:**
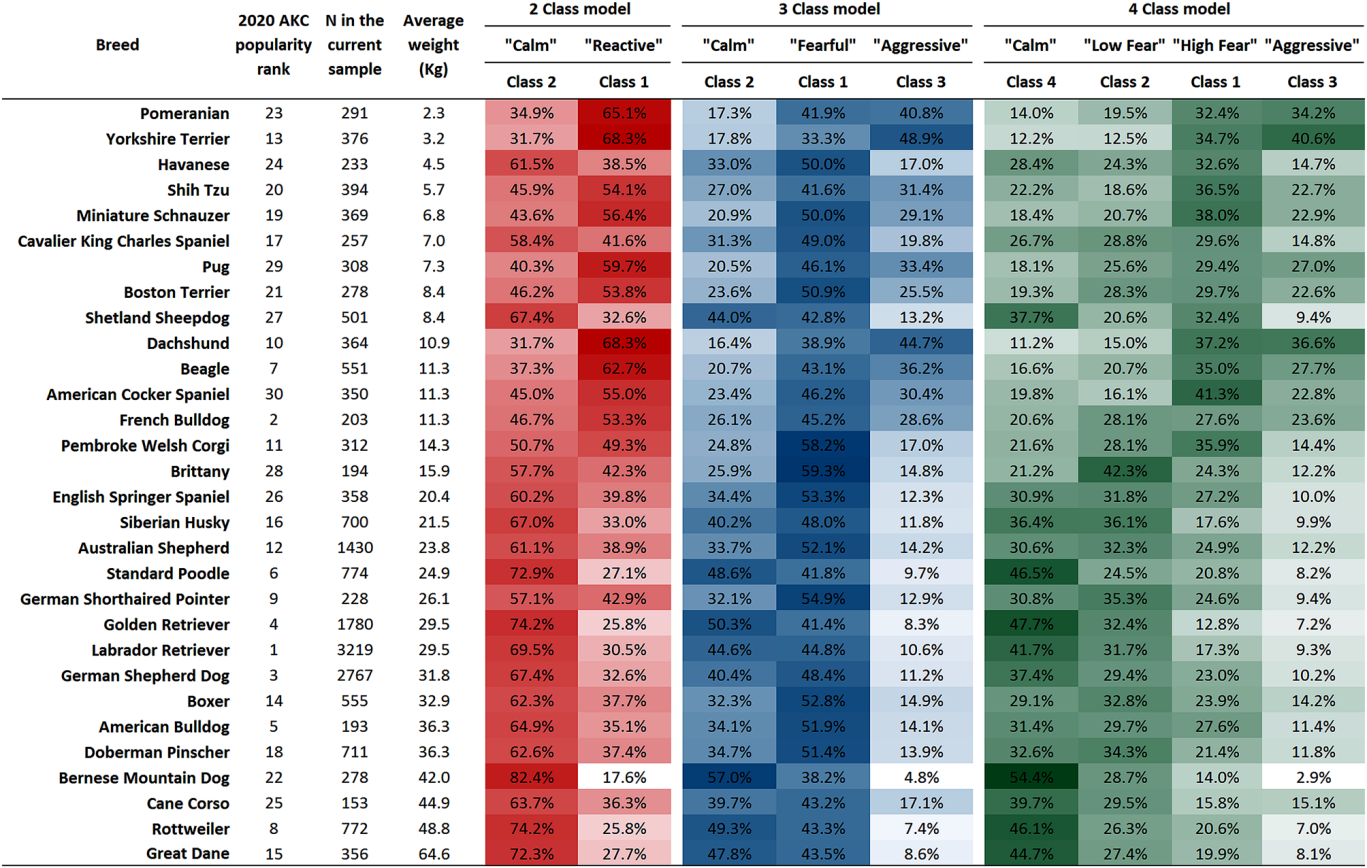
Proportional allocation by class of the top 30 most popular breeds sorted by average weight. Since class numbering is arbitrary by default, it was rearranged by desirability for this figure. Most desirable class is arranged left to right. Each class was summarized by a subjective adjective interpreted from the profiles they displayed.

The C-BARQ LCA approach we present can further be used as a tool that allows for a comparative evaluation of breeds’ average personality profiles. This comparative scheme is presented in Fig. 4 for the 2-, 3- and 4-class models. It shows the top 30 most popular registered breeds in the US^20^ ordered by body size. The effect of body size is evident as color gradients in all models. That pattern, the trait relationships across classes (as in Fig. 3), and the proportions of dogs across models (Fig. 4) suggest the class structures of the three models are interrelated. On that basis, we use a unified nomenclature for all models. In the 3-class example, we observe breeds like the Bernese Mountain Dog and Rottweiler have a similar proportion of “Calm” to “Fearful” to “Aggressive” ratios with the “Calm” group being the most representative for these breeds. In contrast, several of the smallest breeds like the Dachshund and the Yorkshire Terrier show similar high proportions in the “Aggressive” and/or “Fearful” groups. In the 4-class model, the smallest breeds show high proportions in the “Aggressive” and “High Fear” groups. In summary, our results show several strengths of the LCA method which allows predetermination of the number of class groups in a population and incorporation of diverse types of variables; and, here, indicates the basis for subpopulations in terms of their C-BARQ temperament profiles.

## Discussion

In this study, we used an LCA approach to characterize and evaluate the temperament profiles of dog breeds based on their C-BARQ scores. C-BARQ is one of the most popular owner-directed and well validated tools available for canine behavioral assessment and has been used for many purposes^21–23^. Overall, owner-directed dog behavioral assessments have been shown to be reliable and replicable tools. This LCA approach on C-BARQ provides an advantage to conceptually similar methodologies such as factor analysis or hierarchical clustering methods. In factor analysis the number of dimensional clusters that can be formed is subjective and is often limited by visualization and the context of their interpretation. With LCA, the number of clusters can be predetermined and therefore allow for the evaluation of unobserved groups by forcing the grouping to go beyond what is observable in factor analysis^13^. LCA can be used alone^24^, or together with factor analysis to evaluate the properties of specific items in the assessment^25^. In addition, it provides some practical advantages in estimation accuracy when compared to methods such as k-means and hierarchical clustering methods, but with the additional advantage of predefined numbers of clusters^15,26,27^.

Evident advantages of LCA can be seen in our application to C-BARQ scores. LCA allows us to customize how intricate the interpretations will be. In our example, we present 2-, 3-, and 4- class models, arbitrarily focusing on the 3-class model for our extended evaluations. We could similarly have used the 2- or 4- class models if a simpler or more complex interpretation was desired. We envision this quality of LCA as an important advantage. For example, this predefined grouping can be tuned for optimal incorporation into downstream applications such as genomic association analysis for discovery or predictive purposes.

The rich and diverse sample population used in our study (57,454 participants representing 365 breeds) allowed for an exceptional assessment of breed temperament averages. In our results, we show how the C-BARQ captures an important amount of information, far beyond what simpler demographic or environmental covariates do. Despite the very strong P-values presented in Table 1, demographic variables account for an average of only 3.8% of the total variance, based on log-likelihood values. This highlights the effectiveness of C-BARQ as a type of “psychological” dog assessment tool. It is important to note that we decided to stop in this study at 4 classes despite the model fit statistics suggesting that additional classes could continue to improve the model fit. This was done based on convenience for our envisioned usage of the methodology. The purpose of this study was to showcase the potential application of LCA to simplify a complex battery of behavioral assessments. Our study was not aimed at identifying the optimal number of classes. In humans, temperament is a major component that defines personality^28^ as is an expression of unique learning systems^29,30^ that are likely to be shared with dogs. However, since some aspects of these learning systems require self-awareness, we must be cautious in their interpretation as we are dealing with dogs for which we cannot assess self-awareness as in humans. For that reason, we are careful of not anthropomorphize our interpretation and labeling.

The data presented for the top 30 breeds (Fig. 4) hints at how personality classifications could be exploited: for example, when looking for a breed of a predominant temperament type or when looking for multiple similar breeds. This may be useful for selecting dogs breeds for specific working purposes. The number of groups that is predetermined in the LCA can be set as levels of a behavioral variable in downstream evaluations. In this paper we used C-BARQ traits but in a similar way, we could have directly used the 100 questions from which these traits are derived.

One biological implication of our findings is that body size is associated with the structure of all three LCA models. The general observation that most or all common problem behaviors in dogs are correlated with small body size has been made in both behavioral^3,21,31–33^ and genetic^11,34^ studies. In line with that, canine genetic variations associated with both small body size and problem behaviors have also been found to be associated with brain structure differences detectable by magnetic resonance imaging (MRI)^34^. Related MRI studies showed that as dog body size becomes smaller, the ratio of cortex to subcortex volume decreases^35^. This led to the theory that the smaller a dog is, the less inhibition of subcortex by the cortex it exhibits, implying higher reactivity to diverse stimuli. Further studies are necessary to dissect the relationship of body size and LCA models, but our findings are consistent with previous regression^31^ and hierarchical clustering^33^ analyses that showed similar correlations of C-BARQ factors with body size and studies strongly support each other. Although we are not the first ones to report the association between small dog size and behavior, our study highlights the importance of these size effects in the overall study of behavior. Morphological traits such as body size may have a synergistic genetic and environmental association towards expressed behaviors that may not be possible to evaluate separately.

Limitations of LCA and our approach can be directly linked to the population studied and the quality of the assessment tool evaluated, just as with any other analytical tool. A sample with lower representation will carry inherent limitations in detection power just as a noisier assessment tool would. On a technical note, limitations in the LCA approach can also be overcome with simultaneous analyses by other methodologies such as factor analysis or hierarchical clustering. As for any other classification approach, it will be necessary to validate the groupings and demonstrate their utility. Whereas classification involves latent factors, one interesting possibility is that genetic mapping and comparative human behavioral genetics could reveal etiological clues about canine LCA classes. Another approach we favor is the inclusion of diverse, external data, such as by using single-breed birth cohorts of working dogs with standardized environmental, health, training, and behavioral data. We anticipate LCA will complement standard approaches to elucidate the etiology of behavior with implementations such as the temperament assessment we present.

In summary, LCA is a tool that can be exploited to enhance, and is also complementary to, standard approaches. Here, we showed how LCA effectively classifies the C-BARQ assessments of a large population of diverse dog breeds into groupings that can be predetermined according to convenience. In every modality, these groups can be further dissected to define their characteristics such as individual C-BARQ traits. Furthermore, these traits can be ranked in order of desirability which can be introduced as levels for simplified variables of complex constructs, such as temperament. We thus propose these approaches can be used for genetic studies that could lead to improved understanding of the physiological and environmental factors of personality and psychopathology.

## Materials and methods

### C-BARQ data

The data sample in this study consisted of 57,454 CBARQ entries collected through the University of Pennsylvania online portal from 2006 to 2019 (https://vetapps.vet.upenn.edu/cbarq/). These entries represent 365 breeds including pedigree, “designer-breed”, and mixed breed dogs, among others. Since the survey is freely available without special restrictions, our data sampling is considered self-selected. The C-BARQ questionnaire consists of 100 questions in 7 distinct categories: 8 questions related to training, 27 to aggression, 18 to fear, 8 to separation, 6 to excitability, 6 to attachment and attention seeking, and 27 to miscellaneous. Some of the C-BARQ traits were extracted by factor analysis of multiple questions while others correspond to single scores for a specific question^3^. For each entry, basic demographic data and dog characteristics were included. Owner consent to participate in this study was granted with the agreement to contribute to the database. All regulatory approvals for the development of the C-BARQ tool and its use for research purposes was managed by the University of Pennsylvania IACUC and IRB boards (IRB exempt). All methods were performed in accordance with the relevant guidelines and regulations.

### Latent class analysis

Anonymized data were evaluated using Latent Class Analysis (LCA) using PROC LCA in SAS which was developed by the Methodology Center at Penn State University and published in 2015^36^. CBARQ data was evaluated using the individual questionnaire subscales and miscellaneous items. Models were developed for each set with three alternatives 2 class, 3 class and 4 class. In our analysis, we display results for all models but we focus our discussion on the results of the 3-class model. Models incorporated the C-BARQ item scores as ordered multinomial variables (5 levels); therefore, scores for traits with decimal values were rounded to the nearest integer. These correspond to traits that have compounded scores based of several questions, such as fear and aggression traits. Covariates for the models were included as either continuous independent variables (age at evaluations, weight, and age acquired) or binary independent variables (sex, neuter status, health problems, if the dog is the first owned, or other dogs in the household; 0 = Female and 1 = Male for sex and 0 = No and 1 = Yes for all others). For the analysis, we used Rho stabilizing priors of 1 to avoid solutions with Rho estimates of zero or one which would lead to non-estimable Rho values. All analyzes were performed on SAS/STAT v.9.4 (SAS Institute Inc., Cary, NC).

### Temperament profile differences

The ranking of the top 10 traits that would be used to differentiate the class of temperament profiles was performed by calculating the square difference of the item response probabilities (Rho estimates) for each pairwise class comparison for each C-BARQ trait level. (1 vs.2 for the 2-class models; 1 vs. 2, 1 vs. 3, and 2 vs. 3 for the 3-class models; 1 vs. 2, 1 vs. 3, 1 vs.4, 2 vs. 3, 2 vs. 4, and 3 vs. 4 for the 4-class models). This method allowed us to detect the items with the largest probability discrepancies for the data that would point out the most relevant traits for the class. With this ranked trait list, we generated personality profiles that would characterize each class for each model.

## Supplementary Information

Supplementary Information.

## Data Availability

The data that support the findings of this study are available from C-BARQ but restrictions apply to the availability of these data, which were used with their permission for the current study, and so are not publicly available. Data are however available from the authors upon reasonable request and with permission of C-BARQ.
