# Supplementary material for: Latent class analysis of behavior across dog breeds reveal underlying temperament profiles
Source: Sci Rep. 2022 Sep 17;12:15627. doi: 10.1038/s41598-022-20053-6 (PMC9482611; doi:10.1038/s41598-022-20053-6)

Supplementary for

**Latent Class Analysis of behavior across dog breeds reveal underlying temperament profiles**

By

Isain Zapata, Alexander Eyre, Carlos E. Alvarez & James A. Serpell

**Supplementary Table 1.** Fit indices for Latent Class Analysis of C-BARQ traits and questionnaire with and without covariates. Sample size 57,454 dogs.


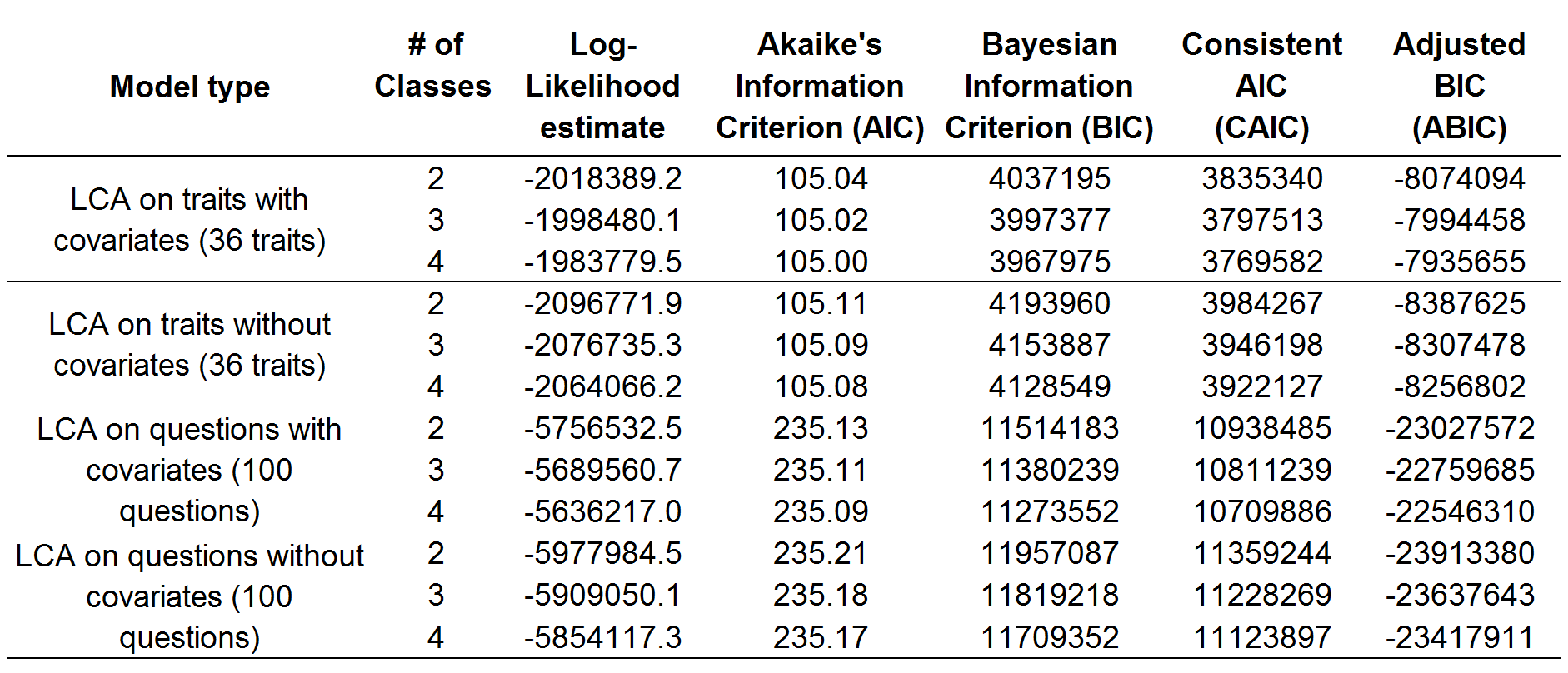

Supplement: Supplementary file 1 — Supplementary Information. [file 41598_2022_20053_MOESM1_ESM.docx]
